# *Pasteurella pneumotropica* Evades the Human Complement System by Acquisition of the Complement Regulators Factor H and C4BP

**DOI:** 10.1371/journal.pone.0111194

**Published:** 2014-10-27

**Authors:** Alfredo Sahagún-Ruiz, Adriana Patricia Granados Martinez, Leandro Carvalho Dantas Breda, Tatiana Rodrigues Fraga, Mónica Marcela Castiblanco Valencia, Angela Silva Barbosa, Lourdes Isaac

**Affiliations:** 1 Departamento de Microbiología e Inmunología, Facultad de Medicina Veterinaria y Zootecnia, Universidad Nacional Autónoma de México, Mexico City, Mexico; 2 Departamento de Imunologia, Instituto de Ciências Biomédicas, Universidade de São Paulo, São Paulo, Brazil; 3 Laboratório de Bacteriologia, Instituto Butantan, São Paulo, Brazil; University of Kentucky College of Medicine, United States of America

## Abstract

*Pasteurella pneumotropica* is an opportunist Gram negative bacterium responsible for rodent pasteurellosis that affects upper respiratory, reproductive and digestive tracts of mammals. In animal care facilities the presence of *P. pneumotropica* causes severe to lethal infection in immunodeficient mice, being also a potential source for human contamination. Indeed, occupational exposure is one of the main causes of human infection by *P. pneumotropica*. The clinical presentation of the disease includes subcutaneous abscesses, respiratory tract colonization and systemic infections. Given the ability of *P. pneumotropica* to fully disseminate in the organism, it is quite relevant to study the role of the complement system to control the infection as well as the possible evasion mechanisms involved in bacterial survival. Here, we show for the first time that *P. pneumotropica* is able to survive the bactericidal activity of the human complement system. We observed that host regulatory complement C4BP and Factor H bind to the surface of *P. pneumotropica*, controlling the activation pathways regulating the formation and maintenance of C3-convertases. These results show that *P. pneumotropica* has evolved mechanisms to evade the human complement system that may increase the efficiency by which this pathogen is able to gain access to and colonize inner tissues where it may cause severe infections.

## Introduction

The genus *Pasteurella* comprises a group of Gram negative bacteria that are commensal and opportunistic pathogens. They affect mainly the respiratory and genital tracts but can also cause systemic infections, responsible for relevant diseases in domestic and wild animals, including, fowl cholera, hemorrhagic septicemia and pneumonia in cattle, swine atrophic or purulent rhinitis in rabbits [1]. *Pasteurella multocida* was one of the first pathogenic bacteria studied and the first live vaccine ever produced from attenuated bacteria [2]. *P. multocida* may be present in the normal bacterial flora residing within the intestinal, respiratory and genital mucosa of various animals. Another species of the same genus, *P. pneumotropica*, is widely encountered as part of the normal flora of the upper respiratory tract of laboratory animals where it is considered an opportunistic pathogen capable of causing pulmonary lesions [3]. It may also cause abortion, and it has been isolated from the lung and spleen of infected mouse fetuses [4].

One of the main sources of human contamination by *Pasteurella* spp is occupational exposure, although infection may also be associated with pet bites and scratching. The clinical presentation of the disease includes cellulitis, subcutaneous abscesses, respiratory tract colonization and systemic alterations [5]. In addition, specific reports of humans infections by *P. pneumotropica* include a wide variety of clinical findings, such as: left-sided and right-sided endocarditis [6], [7]; pneumonia in patients with α anti-trypsin deficiency [8] and HIV [9]; meningitis [10], osteomyelitis and arthritis associated with dog bites [11]; septicemia in an elderly woman living with cats [12]; peritonitis in a patient receiving dialysis [13]; and epidural abscess [14].

Given the ability of *P. pneumotropica* to fully disseminate in the organism, it is plausible that it may have evolved different mechanisms to escape from the attack of the immune system. The complement system plays an important role in innate and acquired immunity. This system can be activated by three independent pathways: the classical, the alternative and the lectin pathways. Once activated, several important biological functions contribute towards pathogen elimination, including: *a)* production of opsonins (fragments iC3b, C3b, C3d and C4d) that enhance phagocytosis; *b)* release of chemotactic factors (fragments C3a and C5a) that attract inflammatory cells to the activation site; *c)* production of anaphylatoxins that trigger mast cell and basophil degranulation (fragments C3a, C4a and C5a) releasing inflammatory mediators; *d)* cell lysis caused by the membrane attack complex (C5b-9_n_); *e)* proliferation of B lymphocytes and increased antibody production (fragment C3d and complement receptor-2); and, *f)* generation of fragment C3d which acts as an adjuvant, reviewed [15].

To prevent activation on self-cells and excessive consumption of complement proteins, several regulatory molecules are present in a soluble form in the plasma or as part of the host cell membrane. Factor H (FH) regulates the alternative pathway (activated independently of antibodies) and C4b-binding protein (C4BP) regulates both the classical (antibody-dependent) and the lectin (innate immunity) pathways [16], [17]. These regulatory proteins are present at relatively high concentrations in plasma (FH = 442.7±105.8 µg/ml, and C4BP = 334.6±82.6 µg/ml) [18]. FH and C4BP act as co-factors of the serine protease Factor I (FI) in the cleavage of C3b and C4b, respectively. They can also accelerate C3-convertase decay (C3bBb for the alternative pathway and C4b2a in the classical and lectin pathways) [15]–[21]. In this way, FH and C4BP are soluble regulators that prevent a continued cascade activation of the three complement pathways. The acquisition of FH and C4BP by human pathogens constitutes an important evasion mechanism, since it blocks the complement activation on their surfaces [22]–[24].

Studies on the role of complement to eliminate bacteria of the genus *Pasteurella* performed to date have employed *P. multocida*. It has been demonstrated that complement resistant strains are more virulent than susceptible ones. Snipes and Hirsh [25] reported a higher susceptibility of an acapsular mutant (P1059-1 A) of *P. multocida* to lysis mediated by complement proteins present in turkey plasma when compared to that observed for the parental P1059-1 strain. Hansen and Hirsh [26] subsequently observed that resistance of *P. multocida* to turkey serum was associated with encapsulation, since non-encapsulated strains and bacteria cultured in media with hyaluronidase were susceptible to complement lysis. Consumption of complement was observed in serum after incubation with either encapsulated or non-encapsulated bacteria. It was therefore suggested that in spite of activation of the complement system on the bacterial surface, the capsule somehow acts as a shield against the insertion of the membrane attack complex into the outer membrane [26], a mechanism that is distinct from one that would work via inhibition of complement activation. On the other hand, the contribution of the capsule in complement resistance is not completely clear: Boyce and Adler [27] constructed a *P. multocida* mutant impaired in capsule synthesis that was just as resistant to complement bovine serum proteins as the wild type strain. However, the acapsular *P. multocida* mutant was more susceptible to phagocytosis than the wild type. Blau *et al* [28] observed that clinical isolates of *P. multocida* or *P. haemolytica* (now *Mannheimia haemolytica*) varied considerably in their resistance or susceptibility to lysis by fresh bovine serum. Serum sensitivity of *P. multocida* was observed even in the presence of EGTA-MgCl_2_ (conditions in which the classical and lectin pathways are not activated) indicating that the alternative pathway was likely the most important pathway activated by this pathogen.

Despite all these studies with *P. multocida*, to our knowledge, the role of the complement system in *P. pneumotropica* has not yet been investigated. This work aimed to analyze the survival of *P. pneumotropica* when incubated with non-immune human serum. Here we investigated a possible evasion mechanism employed by this bacterium to evade complement, by the acquisition of negative complement regulators (FH and C4BP) which could contribute to the bacterial survival in the host, and favor the development of disease.

## Materials and Methods

### Ethics statement

Protocols were previously approved by the local institutional committee. (06/10/CEUA/ICB) from the Institute of Biomedical Sciences, University of São Paulo. The bacteria *P. pneumotropica* used in this study were accidentally isolated as a contaminant present in a suspension of leptospires which originally came from one single hamster. We did not perform experiments with animals in this study. A pool of normal human serum from anonymous adult healthy donors was obtained after informed and written consent signed by each individual. The Ethics Committee on Research on Humans of the Institute of Biomedical Sciences of the University of São Paulo has approved the use of the human serum samples in this study, according to the memorandum Of. CESH.023.12. The human serum samples were anonymized before use.

### Bacteria

*P. pneumotropica* was isolated from a hamster liver at the Institute of Biomedical Sciences, University of São Paulo, and identified using the API system at the Butantan Institute, São Paulo, Brazil. The hamster was anesthetized with ketamine (115 mg/kg) and xylazin (10 mg/kg) before being sacrificed in a CO_2_ chamber.

Glycerol (40%) aliquots of *P. pneumotropica* were prepared from overnight Luria Bertani (LB) broth cultures from a single colony on LB agar, and kept at −80°C. Before use *P. pneumotropica* were thawed and cultured in LB medium for 12–14 h at 37°C under moderate agitation (185 rpm) until the culture reached an optical density between 0.4 to 0.5 at 600 nm (approximately 1.6×10^8^ to 2×10^8^ bacteria/ml) (O.D._600 nm_ 0.25 corresponds approximately to 1×10^8^ bacteria/ml). After centrifugation at 4400×*g* for 15 min at 21°C, the bacterial pellet was washed once with phosphate buffered saline (PBS), pH 7.4.

### Proteins, antibodies and sera

Purified plasma-derived human complement proteins (C3, C3b, C4b, FH, C4BP, FI) and polyclonal goat or rabbit antibodies against them were purchased from Complement Technology (Tyler, Texas, USA). The secondary anti-IgG antibodies conjugated with peroxidase or with fluorescein isothiocyanate (FITC) were purchased from KPL (Tyler, Texas, USA). A pool of normal human sera (NHS) was obtained from healthy volunteers, after informed written and signed consent. All human serum samples used here were anonymized. Heat-inactivated NHS (HI-NHS), incubated at 56°C for 30 min, was used as a negative control of complement activation.

### Bacterial survival in the presence of human serum

After 14 h of growth in liquid media, the number of *P. pneumotropica* was estimated by measuring absorbance (OD_600 nm_) and adjusted to 1×10^3^ bacteria/tube. Subsequently, *P. pneumotropica* was incubated with 40% NHS or HI-NHS at 37°C under moderate agitation (185 rpm). After 30 min of incubation, suspensions were centrifuged and supernatants were removed. The bacterial pellet was suspended in PBS, diluted (2 tenfold dilutions), and spread in LB agar plates (triplicate) and incubated at 37°C for 28 h. The number of colony forming units (CFU) was determined and the percentage of survival was calculated. The number of colonies obtained after incubation with HI-NHS was considered 100% survival.

To evaluate the importance of FH for the survival of *P. pneumotropica* in the presence of complement system, we first incubated the bacterial suspension (approximately 1×10^3^ bacteria/tube) with human purified FH (125, 375 or 500 µg/ml) for 5 min at 37°C before adding 40% of FH-depleted human serum (Complement Technology) followed by 30 min incubation at 37°C. Next, suspensions were centrifuged and supernatants were removed. The bacterial pellet was suspended in PBS and diluted (2 tenfold dilutions) before spreading in LB plates (triplicate). The number of colonies was counted after 28 h incubation at 37°C. A negative control was included containing no purified FH before treating *P. pneumotropica* with FH-depleted serum.

### Quantification of complement proteins bound to *P. pneumotropica* by ELISA

To analyze C3b, FH or C4BP deposition, *P. pneumotropica* suspensions (2×10^8^ bacteria/tube) were incubated for 30 min or 60 min with 20% NHS, 20% HI-NHS or PBS, at 37°C under agitation (185 rpm). After centrifugation (5500×*g* for 10 min), bacterial pellets were washed three times with PBS, suspended in 100 mM sodium carbonate (pH 9.6) and immobilized in microtiter polystyrene plates (Costar, High Binding) for 16 h at 4°C. The plates were washed twice with PBS-T (PBS pH 7.4 containing 0.05% Tween-20) after each one of the following steps. The nonspecific binding sites were blocked with 3% BSA in PBS for 2 h at 37°C. The plates were then incubated with primary antibodies: goat anti-human C3 (diluted 1∶5000), goat anti-human FH (diluted 1∶10000), rabbit anti-human C4BP (diluted 1∶2000), for 1 h at 37°C. Subsequently, the secondary antibodies were added: anti-goat IgG-peroxidase conjugated or anti-rabbit IgG peroxidase-conjugated (both diluted 1∶10000). Ortho-phenylenediamine dihydrochloride (0.004%) in citrate-phosphate (pH 5.0) containing 0.015% H_2_O_2_ was used as substrate. The reaction was stopped with 50 µl of H_2_SO_4_ 2 M/well. As negative controls, we used untreated bacterial suspensions but similarly incubated with the respective primary and secondary antibodies. Absorbances at 492 nm were taken using an ELISA reader (Molecular Devices Spectra Max Plus^384^).

### Flow cytometry analysis

After 14 h of incubation, bacterial cultures were centrifuged at 5500×*g* for 15 min at 4°C and the pellets were suspended in 20 ml PBS (pH 7.4). Suspensions containing *P. pneumotropica* (2×10^8^ bacteria/tube) were incubated for 2 h with purified FH (1 µg), C4BP (1 µg) or for 30 min with 20% NHS at 37°C. Bacteria were washed with PBS and incubated for 30 min with polyclonal goat anti-human FH or polyclonal rabbit anti-human C4BP (diluted 1∶100) in PBS. After washing, *P. pneumotropica* suspensions were incubated for 30 min, rabbit anti-goat IgG-FITC conjugate (diluted 1∶200) or goat anti-rabbit IgG-FITC conjugate (diluted 1∶200) in PBS. As negative controls, untreated *P. pneumotropica* suspensions incubated only with primary and secondary antibodies were used. Bacteria were detected using log-forward and log-side scatter dot-plot. The gate was drawn to exclude debris and larger aggregates of bacteria - 10,000 bacteria/events were captured per experiment. FH and C4BP binding were measured by geometric mean fluorescence intensity (GMFI) on FACS Canto II (Benton Dickson). The data were analyzed by FlowJo Software (version 8.7).

### Cofactor activity assays

The functional regulatory activity of FH or C4BP bound to *P. pneumotropica* as a co-factor for FI was analyzed. Bacteria were collected by centrifugation (5500×*g* for 15 min, at 21°C), suspended in 20 ml PBS (pH 7.4). Suspensions containing 2×10^8^ bacteria/tube were incubated for 1 h at 37°C with FH (1 µg), C4BP (1 µg) or 10% NHS in a final volume of 500 µl. Bacteria were then washed three times with PBS and C3b and FI (0.5 µg of each) were added to the bacterial suspensions pre-treated with FH, 10% NHS or PBS, while C4b and FI (0.5 µg of each) were added to the bacterial suspensions pre-incubated with C4BP, 10% NHS or PBS. These suspensions were then incubated at 37°C for 1, 2 or 4 h. The functional cofactor activities were evaluated by Western blot, and the cleavage fragments of C3b and C4b were detected using goat polyclonal primary anti-human C3 or goat anti-human C4 (diluted 1∶10000) antibodies and secondary anti-goat IgG-peroxidase conjugated or anti-rabbit IgG-peroxidase conjugate antibodies.

### Statistical analysis

Data were analyzed using Student’s *t*-test or ANOVA and *p* values are indicated at each figure legend.

## Results

### *Pasteurella pneumotropica* survives complement system activation

**Figure 1:**
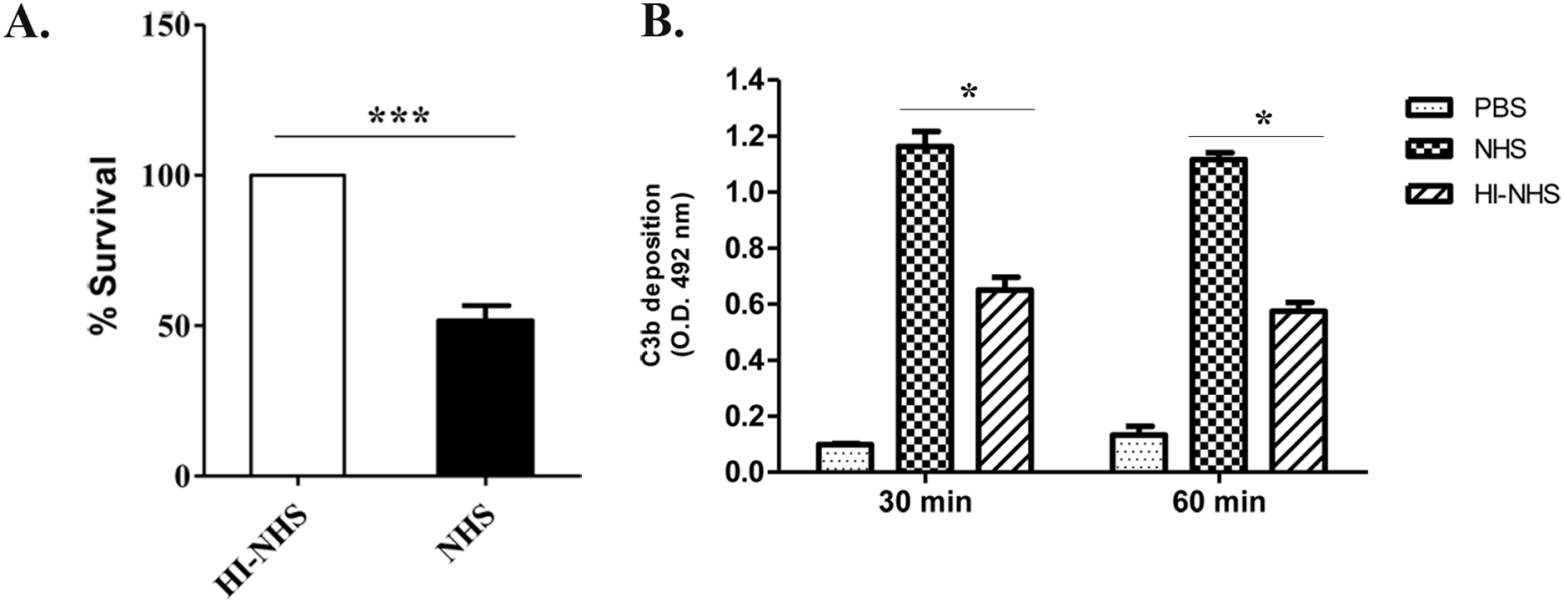
*P. pneumotropica* is resistant to the complement system action. (**A**) *P. pneumotropica* suspensions (10^3^ bacteria/tube) were incubated with 40% of normal human serum (NHS) or heat inactivated (HI-NHS) diluted in PBS pH 7.4, at 37°C for 30 min and then sera was removed and bacteria suspended in PBS, diluted (2 tenfold dilutions) and cultivated in LB agar plates (triplicate) at 37°C for 28 h. Data are expressed as percentage of colony forming units, considering HI-NHS as 100%. The data obtained from ten independent experiments is shown. (**B**) C3b binding to *P. pneumotropica.* Bacteria suspensions (10^8^ bacteria/tube) were incubated for 1 h at 37°C with 20% NHS or HI-NHS. After washing, C3b deposition was quantified by ELISA with anti-C3 polyclonal antibodies. Data were obtained from three independent experiments and analyzed statistically using Student’s *t*-test where *p<0.05; ***p<0.0001.

The effect of the complement system in *P. pneumotropica* was assessed upon incubation of the bacteria culture with 40% non-immune NHS for 30 min. The percentage of surviving cells was calculated by comparing the number of viable bacteria incubated in NHS with those incubated in HI-NHS (considered 100%). The results indicate that *P. pneumotropica* is able to resist complement system activation (**Figure 1A**). In ten independent experiments approximately half of viable bacteria were detected after 30 min of incubation. Complement system activation was also assessed by evaluating C3b deposition on the surface of *P. pneumotropica* after incubation with NHS and HI-NHS by ELISA (**Figure 1B**). C3b deposition was markedly increased upon incubation in NHS.

### *Pasteurella pneumotropica* acquires complement regulators from human serum

**Figure 2:**
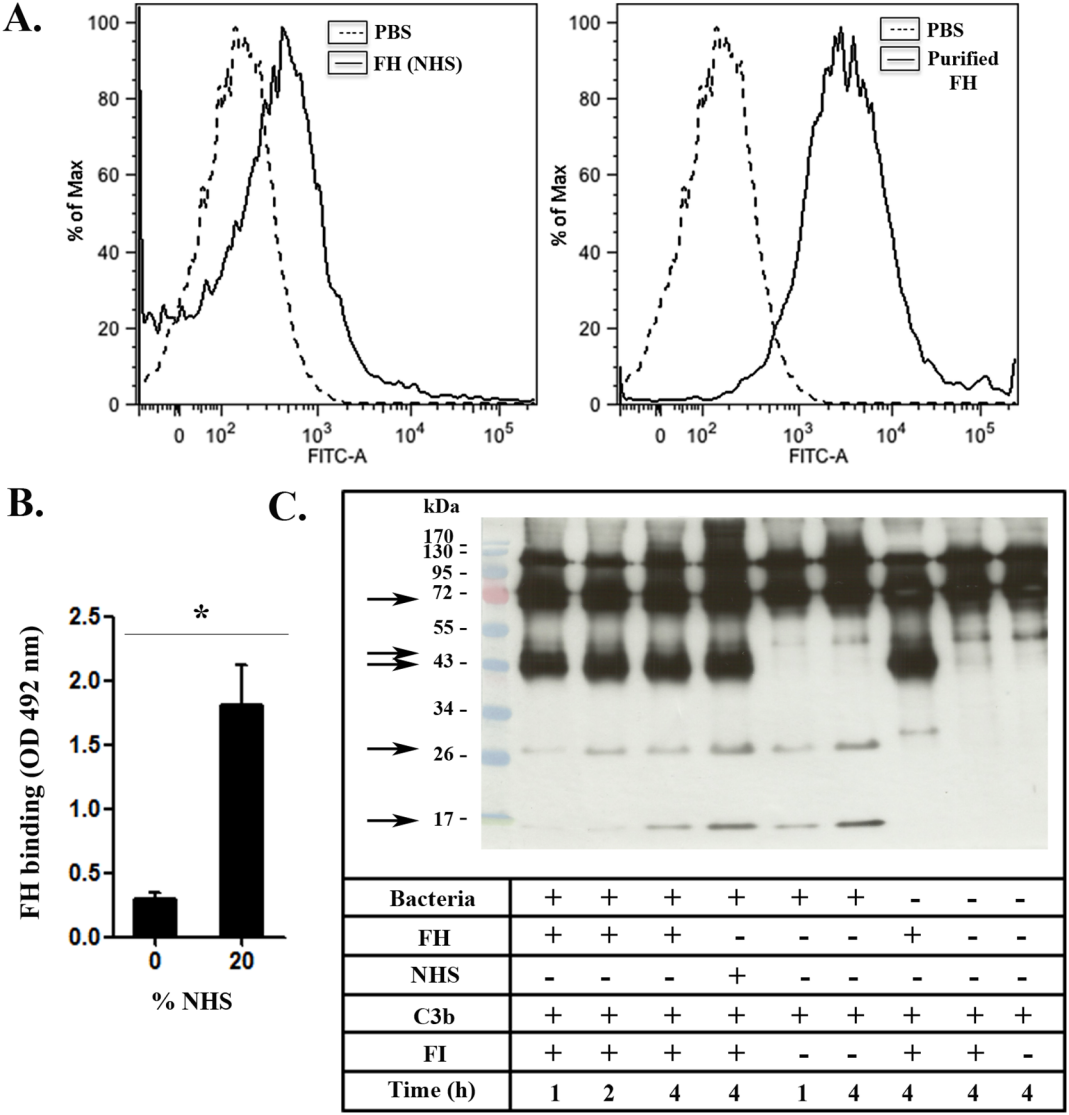
*P. pneumotropica* binds to Factor H and regulates the alternative complement pathway. (**A**) *P. pneumotropica* suspensions (10^8^ bacteria/tube) were incubated with 1 µg of human Factor H (FH) for 2 h or normal human serum (NHS) for 30 min. After washing, bound FH was detected by incubation with goat polyclonal antibody anti-FH (primary antibody) and then with anti-goat IgG-FITC conjugated (secondary antibody) (line histogram). As a negative control (left peak), untreated *P. pneumotropica* was incubated only with primary and secondary antibodies (dashed histogram). (**B**) Serum FH binding to *P. pneumotropica* was quantified by ELISA (*p<0.01). (**C**) Suspensions containing 2×10^8^ bacteria/tube were incubated for 1 h at 37°C with FH (1 µg), or 10% NHS and then washed three times with PBS. C3b and FI (0.5 µg of each) were added to the bacterial suspensions pre-treated with FH, 10% NHS or PBS. These suspensions were then incubated at 37°C for 1, 2 or 4 h. The functional cofactor activity was evaluated by Western blot, and the cleavage fragments of C3b were detected using goat polyclonal primary anti-human C3. FH bound to *P. pneumotropica* acts as a co-factor of Factor I (FI) for the C3b cleavage. C3b cleavage fragments are indicated by arrows. Negative controls were included to observe spontaneous C3b cleavage in the absence of FH and/or FI.

According to the results presented above, *P. pneumotropica* is able to survive the bactericidal activity of the activated complement system (**Figure 1**). This fact indicates that *P. pneumotropica* may have evolved evasion mechanisms that allow its survival in NHS. Considering this possibility, we decided to evaluate if the host negative complement regulators FH and C4BP could bind to *P. pneumotropica* and thereby confer protection from complement-mediated lysis. To this aim, bacteria were incubated with purified proteins (FH or C4BP), NHS (as a source of FH or C4BP) or PBS (negative control). In this assay, we verified that *P. pneumotropica* was able to interact with the complement purified proteins FH and C4BP when analyzed by flow cytometry (**Figures 2A** **and** **3A, left panel** **s**). Moreover, the interaction of *P. pneumotropica* with these regulators also occurred when NHS was used as a complement source, as observed by flow cytometry (**Figures 2A** **and** **3A, right panel** **s**) and ELISA (**Figure 2B** **and** **3B**).

**Figure 3:**
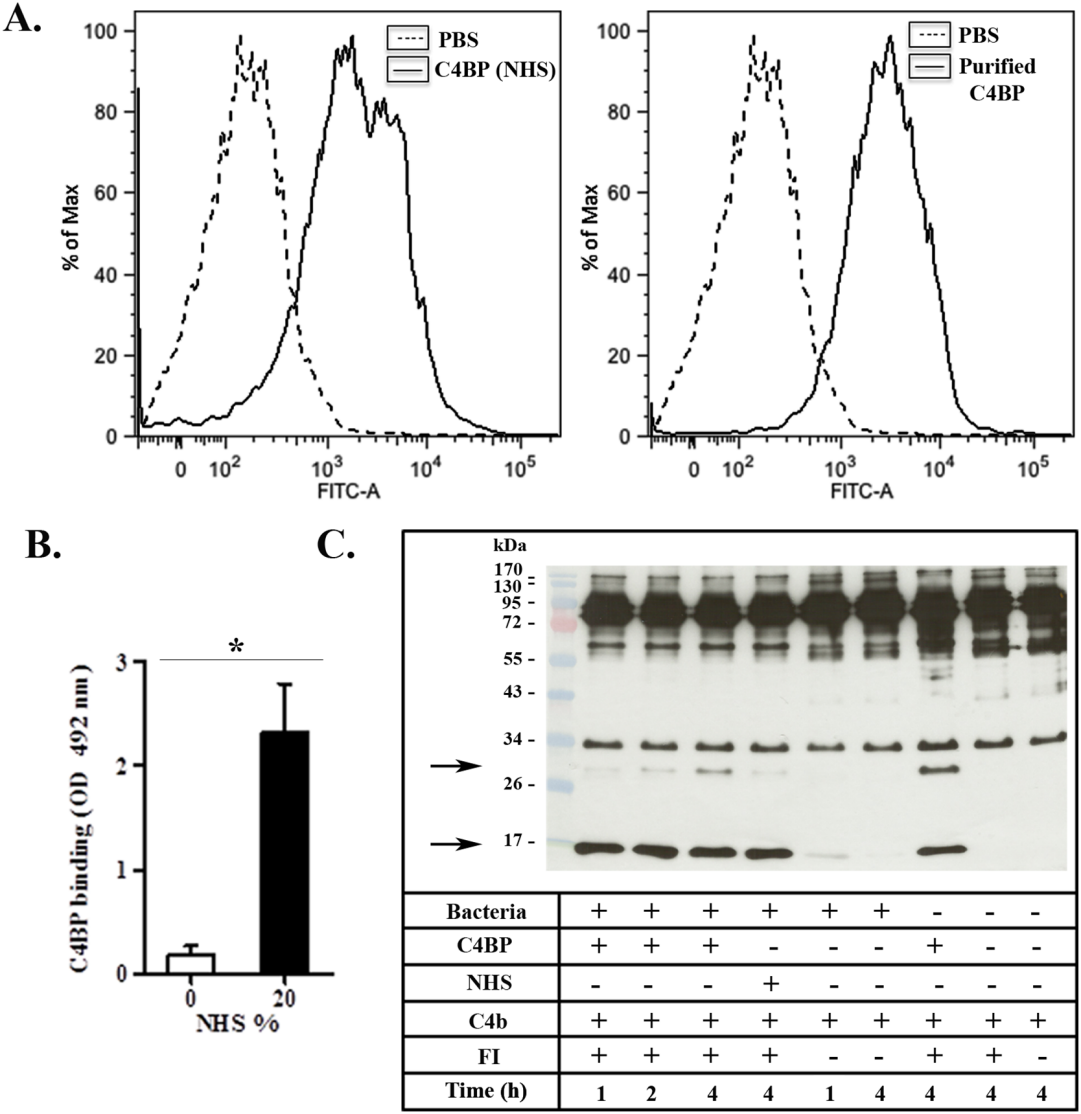
*P. pneumotropica* binds to C4b-binding protein and regulates the activation of the complement classical and lectin pathways. (**A**) *P. pneumotropica* (10^8^ bacteria/tube) was incubated with human C4-binding protein (C4BP) (1 µg) for 2 h or with normal human serum (NHS) for 30 min, followed by incubation with goat polyclonal antibody anti-C4BP (primary antibody) and then with anti-goat IgG-FITC conjugated (secondary antibody) (line histogram). As a negative control, untreated *P. pneumotropica* was incubated only with primary or secondary antibodies (dashed histogram). (**B**) Serum C4BP binding to *P. pneumotropica* was quantified by ELISA (*p<0.01). (**C**) Suspensions containing 2×10^8^ bacteria/tube were incubated for 1 h at 37°C with C4BP (1 µg), or 10% NHS and then washed three times with PBS. C4b and FI (0.5 µg of each) were added to the bacterial suspensions pre-treated with C4BP, 10% NHS or PBS. These suspensions were then incubated at 37°C for 1, 2 or 4 h. The functional cofactor activity was evaluated by Western blot, and the cleavage fragments of C4b were detected using goat polyclonal primary anti-human C4. C4BP bound to *P. pneumotropica* acts as a co-factor of Factor I (FI) for the C4b cleavage. C4b cleavage fragments are indicated by arrows. Negative controls were included to observe spontaneous C4b cleavage in the absence of C4BP and/or FI.

### FH and C4BP retain cofactor activity when bound to *P. pneumotropica*

To assess the functional activity of *P. pneumotropica* surface-bound FH and C4BP, whole bacteria were incubated with NHS, or with purified FH or C4BP. After washing, C3b or C4b and FI were added. The cleavages of C3b or C4b were analyzed by Western blotting using anti-human C3 or anti-human C4 polyclonal antibodies. Control reactions included one positive control with only the purified proteins (C3b, FH and FI) without bacteria; and four negative controls: a) bacteria with FI and C3b, b) bacteria with only C3b, c) FI and C3b without bacteria and d) C3b without bacteria. For C4BP similar controls were used, but substituting FH for C4BP and C3b for C4b.

In these assays we observed that FH and C4BP acquired by *P. pneumotropica* retained their cofactor activities (**Figures 2C** **and** **3C**). Bound C4BP was able to act as a FI co-factor in C4b cleavage, producing fragments with approximately 30 kDa and 16 kDa [29] (**Figure 3C**). In a similar way, FH acquired by the bacteria also acted as a FI co-factor in C3b degradation, generating bands with 67 kDa, 46 kDa and 43 kDa (**Figure 2C**). These degradation products were produced by C3b α’chain (115 kDa) cleavage, as previously described [30]. Because the 67 kDa degradation fragment and the C3b β chain (75 kDa) have similar electrophoretic mobilities, and the 46 kDa band co-migrates with the 43 kDa band in SDS-PAGE this distinction is better visualized when the film was exposed for a shorter period of time. Besides these cleavage products, we also observed additional bands with approximately 28 kDa and 15 kDa. Interestingly, these fragments were also obtained when *P. pneumotropica* was incubated with C3b in the absence of FI and FH. This observation suggests a possible role of bacterial protease(s) in C3b cleavage.

### Acquisition of FH contributes to *P. pneumotropica* survival

To assess the importance of acquired FH for *P. pneumotropica* survival, we incubated the bacterial suspension with purified human FH (125, 375 or 500 µg/ml) and then added 40% FH-depleted human serum. A negative control with no previous incubation with FH before treating *P. pneumotropica* with FH-depleted serum was also included.

**Figure 4:**
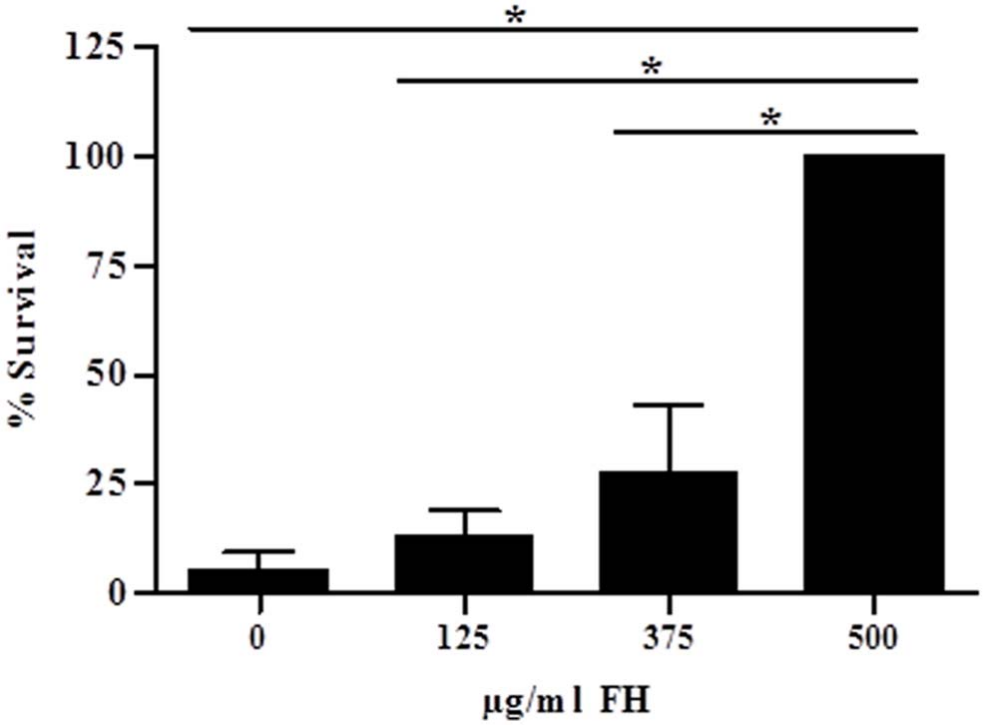
FH is important for *P. pneumotropica* survival in the presence of human serum. Suspensions of *P. pneumotropica* (1×10^3^ cells) were incubated with human purified Factor H (FH) (125, 375 or 500 µg/ml) for 5 min at 37°C and 40% of FH-depleted human serum was added and incubated for 30 min at 37°C. Suspensions were then centrifuged and the bacterial pellet was suspended in PBS and 100×diluted before spreading on LB plates. A negative control was included containing no purified FH before treating *P. pneumotropica* with FH-depleted serum. The percentage of survival was calculated considering 100% as the number of colonies obtained after *P. pneumotropica* treated previously with 500 µg/ml FH before adding FH-depleted serum. Data were analyzed using ANOVA; *p<0.05.

When *P. pneumotropica* was incubated with FH-depleted human serum for 30 min bacterial survival was greatly compromised when compared to NHS (**Figure 4**) confirming the importance of this host regulatory protein for evasion of this pathogen from the immune system. This observation was corroborated when the protection was restored after addition of purified human FH (500 µg/ml) within the physiological range concentration (442 µg/ml±106 µg/ml; reference [18]) to FH-depleted human serum (**Figure 4**). We therefore conclude that survival of *P. pneumotropica* in NHS can be mainly attributed to its interaction with the soluble complement regulator FH of the alternative pathway.

## Discussion

The contribution of the complement system to eliminate bacteria of the genus *Pasteurella* is poorly understood. The studies performed to date have only employed *Pasteurella multocida* [25]–[28] and they demonstrated that complement resistant strains are more virulent than susceptible ones [25]. Boyce and Adler [27] reported that two different strains of *P. multocida* PBA 1514 (encapsulated) and PBA 875 (acapsular mutant) showed a survival of 64% and 42%, respectively, in the presence of normal fresh bovine serum when compared to heat inactivated bovine serum (100%) after 4 h-incubation. In this work, we observe that approximately 50% of *P. pneumotropica* was able to survive when incubated with NHS for 30 min (**Figure 1**). These data prompted us to investigate the possible evasion mechanisms involved in *P. pneumotropica* survival. In this regard, we focused our studies on the acquisition of the host complement regulators FH and C4BP. These regulators were chosen because they are involved in the control of all complement activation pathways: the alternative (FH), the classical and the lectin pathways (C4BP). In addition, several human pathogens bind these regulators to evade the host complement system, reviewed in references [22]–[24].

We observed that *P. pneumotropica* is able to acquire both negative regulators FH and C4BP (**Figures 2A–B** **and** **3A–B**). Once bound to the bacteria surface, FH and C4BP remain functional, since their co-factors activities for FI cleavages of C3b and C4b were preserved (**Figures 2C** **and** **3C**). In this way, the binding of these host regulators to *P. pneumotropica* allow the inactivation of the three complement pathways in the pathogen's surface.

The importance of FH to *P. pneumotropica* survival was further confirmed using FH-depleted serum (**Figure 4**). In this assay, a low number of bacteria was able to survive after incubation with FH-depleted serum for 30 min. Furthermore, the addition of 500 µg/ml of purified human FH (normal range in adult serum: 442 µg/ml±106 µg/ml [18]) to human FH-depleted serum significantly increased the survival of *P. pneumotropica*. Similar mechanisms of complement resistance have been reported for other pathogens such as *Leptospira interrogans* which are able to bind FH and C4BP [31]–[34], *Neisseria meningitidis* that binds human FH through a 33 kDa surface protein and in this way, this regulatory protein retains cofactor activity for FI in C3b cleavage [35], [36]; the spirochetes *Borrelia burgdorferi*, *B. recurrentis* and *B. duttonii,* that are serum resistant because they bind human FH and C4BP [37]–[40], *Haemophilus influenzae* type b that binds FH and FH-like-1 [41], [42], and also *Porphyromonas gingivalis* that binds C4BP through the cysteine protease high molecular weight arginine-gingipain A (HRgpA) which contributes to its serum resistance [43], reviewed in [44]–[46].

According to our data, we can suggest a main role of FH in the survival of *P. pneumotropica* in NHS. Therefore, the identification of possible bacterial ligands for this host regulator is an important issue to be addressed. In this way, it has been reported that *P. multocida* incorporates host sialic acid into its bacterial lipooligosaccharide. Sialic acid is a monosaccharide present on the surface of eukaryotic cells that increases FH affinity for C3b, of the C3-convertase (C3bBb) of the alternative pathway, accelerating its decay which is essential to avoid complement activation on host cells and to regulate the amplification loop of the alternative complement pathway. FH also acts as a co-factor of FI in the cleavage of C3b into iC3b, interrupting the complement cascade activation on the cell surface [20], [21]. *P. multocida* constructed a mutant in sialic acid uptake resulted in attenuation at challenge in the turkey infection model, although both this mutant and the parental strain were equally resistant to killing by turkey serum, in contrast to an acapsular mutant (ΔhyaE) that was highly susceptible to complement [47]. Considering these findings, it would be quite relevant to investigate if *P. pneumotropica* is also able to uptake host sialic acid and then verify its possible role in FH binding and complement resistance.

Despite the evident role of FH in *P. pneumotropica* survival in serum, we cannot completely exclude the participation of other evasion mechanisms, such as the binding of C4BP and the activity of bacterial proteases. Indeed, in the C3b cleavage assay (**Figure 2C**) we observed FI-independent cleavage products, which might indicate a possible endogenous proteolytic activity of *P. pneumotropica*. Moreover, in this study we have also evaluated the C4BP binding to *P. pneumotropica* (**Figure 3A–B**). C4BP bound to the pathogen was able to act as cofactor of FI in C4b cleavage (**Figure 3C**). Therefore, we can speculate that these mechanisms could act together to favor bacteria survival in human serum, but the real contribution of C4BP and proteases must be better explored in *P. pneumotropica.*
